# Experimental Investigation of the Failure Scenario of Various Connection Types between Thin-Walled Beam and Sandwich Panel

**DOI:** 10.3390/ma15186277

**Published:** 2022-09-09

**Authors:** Katarzyna Ciesielczyk, Robert Studziński

**Affiliations:** Institute of Building Engineering, Poznan University of Technology, Piotrowo 5 Street, 60-965 Poznan, Poland

**Keywords:** sandwich panels, thin-walled beam, failure scenario, connections, laboratory experiment

## Abstract

The paper presents failure scenarios for various types of connections between a thin-walled beam and a sandwich panel. In addition to standard connections used in civil engineering applications, that is, self-drilling fasteners for sandwich panels, the study examined the use of bolts, blind rivets, and double-sided acrylic tape applied linearly and pointwise. The connections were subjected to the horizontal load applied with constant eccentricity with respect to the plane of the connection surface. This load arrangement simulates the behaviour of a free flange of the thin-walled beam in bending while lateral-torsional buckling occurs. In this way, the research covers the determination of the lateral stiffness of the thin-walled beam-free flange, while the other flange is connected to the sandwich panel using various connection systems.

## 1. Introduction

Claddings are inseparable elements of industrial halls. The purpose of the claddings is three-fold. Firstly, the claddings protect the structure of the building from environmental actions such as wind, rain, and snow [1]. Secondly, the claddings ensure the desired temperature and moisture content in the interior of the building [2]. Thirdly, the claddings transmit the mechanically (dead load, climatic action, and useful loads) and thermally (gradient temperature between the facings) induced loads to the main or secondary structural elements [3]. Nowadays, the cladding systems in steel industrial halls are increasingly made of sandwich panels [4]. The sandwich panel is a composite element consisting of the two external facings and the internal core [5]. In civil engineering applications, the facings are made of high strength and of a high stiffness material [6,7,8] such as steel, aluminium, or less frequently composite laminate. Their thickness is approximately 0.5 mm in the case of metallic facings and 0.7 mm in the case of laminate facings. The core, from a mechanical point of view, can be defined as a soft and flexible material of low weight and excellent thermal insulation properties. In building construction, the core, which is usually made of mineral wool, polyurethane foam, or styrofoam layer, is significantly thicker than the facings. Its thickness ranges from 40.0 mm to 200.0 mm (depending on the desirable level of thermal insulation). Usually, the core is assumed to be isotropic; nevertheless, recent research [9,10] also investigates its anisotropy.

Sandwich panels are usually attached to the main structural elements and secondary elements (hot-rolled or thin-walled cross-sections) via self-drilling fasteners. The size and type of load transferred by the fasteners (from surface load to the linear load) depend on the assumed ‘structural class’ according to Eurocode 3 [11] associated with the failure consequences defined in Eurocode 0 [12]. Structural classes provide information on the reliability level of cold-formed elements (thin-walled beams) and claddings (corrugated metal plates). The authors of this paper, according to the guidebook titled European Recommendations on the Stabilization of Steel Structures by Sandwich Panels [13], published by the European Convention for Constructional Steelwork, assumed that structural classes can be extended to structures with sandwich panels used as cladding. The mentioned document [13] is the basis for the new standard in development prEN 14509-2 [14]. According to Eurocode 3 [11], three construction classes are distinguished. Construction class I refers to construction where cold-formed members and cladding are designed to contribute to the overall strength and stability of a structure. Construction class II refers to construction where cold-formed members and cladding are designed to contribute to the strength and stability of individual structural members. Construction class III refers to the construction in which the cladding is used as an element that only transfers loads to the main structure. From a mechanical point of view, the fasteners in each construction class participate in transferring the surface load from plate elements (claddings) to their supports, i.e., beam elements. Depending on the direction of the support load in relation to the support plane and the angle of its inclination in relation to the ground plane, the fastener is subjected to axial and/or shear force. Note that compression is mainly transferred from the cladding element directly to the beam flange due to the contact surface. However, in the case of construction classes I and II, it is taken into account that the fasteners transfer the additional membrane forces (forces perpendicular to the fastener axis) that arise from the lateral-torsional stabilization provided to the thin-walled beam elements by the claddings [15,16,17,18]. Additionally, in [19], the hot-rolled sections were considered as beam elements stabilized by sandwich panels. Nowadays, this type of interaction is often used by structural engineering designers in their analyses. Please note that the connection mechanical properties (stiffness, ultimate capacity, and deformation capacity) are also important in other structural elements used in building applications. For example, in [20,21] the authors investigated the various connections in the aluminium–timber composite beam (ATC) that consist of the aluminium beam and laminated veneer lumber (LVL). 

The experimental research presented in this article simulates the behaviour of the free flange of the thin-walled Z beam in bending. The other flange is laterally restrained by the sandwich panel. This laboratory model refers to the calculation method described in Section 10.1 of Eurocode 3, Parts 1–3 [11], where the free flange of the thin-walled beam is considered as a beam on an elastic foundation. The research covers the determination of the lateral stiffness of the thin-walled beam-free flange, while the other flange is connected to the sandwich panel using various connection systems: self-drilling fasteners for sandwich panels, blind rivets, bolts, and double-sided acrylic tape. In addition, the equilibrium path (force-displacement), initial and secant stiffness, ultimate capacity, and deformation capacity were determined. Furthermore, the research allows determining failure scenarios of various connection types between a thin-walled beam and a sandwich panel. 

Currently, as part of the work of the ECCS working group TWG 7.9 named Sandwich Panels and related Structures, the new document is being prepared. This document covers the experimental aspects of the determination of the rotational restraint provided by sandwich panels. The ECCS document, among others, was prepared on the basis of the following articles [22,23]. The laboratory tests presented in the manuscript are in line with the scope of this document, in which the sandwich panel can provide rotational restraint for thin-walled elements by means of various types of connector.

Note that in the research, the thin-walled beams used in the experiments were without openings. The results can be easily extended, for example, on the thin-walled beams with perforations discussed in References [24,25]. In these papers, the authors proposed a method for determining the equivalent stiffness of beam with and without openings or perforations along its length. To be specific in the presented homogenization method, there is no need to provide formal analysis such as solving the system of equations. The method uses the 3D representation of a beam modelled with shell finite elements and global stiffness matrix of the representative volumetric element (RVE).

## 2. Materials and Methods

### 2.1. Problem Formulation

**Figure 1:**
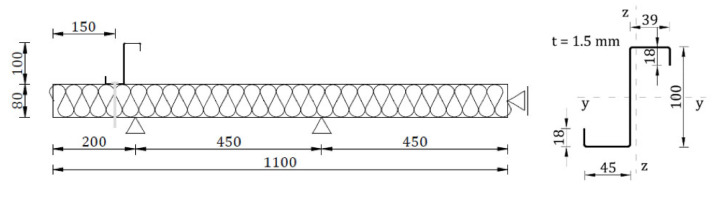
Test bed scheme—geometry of the sandwich panel and the thin-walled Z-beam.

In Figure 1, the scheme of the test bed is depicted. The test bed consisted of one sandwich panel with the following dimensions: width 1100 mm, length 1100 mm, and thickness 80 mm. The sandwich panel was made of two steel facings with a thickness equal to: 0.545 mm (external–upper) and 0.491 mm (internal–bottom) and a soft core made of polyisocyanurate foam (PIR). The mechanical properties of the sandwich panel layers were determined by previously conducted research. Young’s modulus of facings was equal to 190.0 MPa [26], and the value of the shear modulus of the polyurethane foam core was equal to 3.43 MPa [27]. 

**Figure 2:**
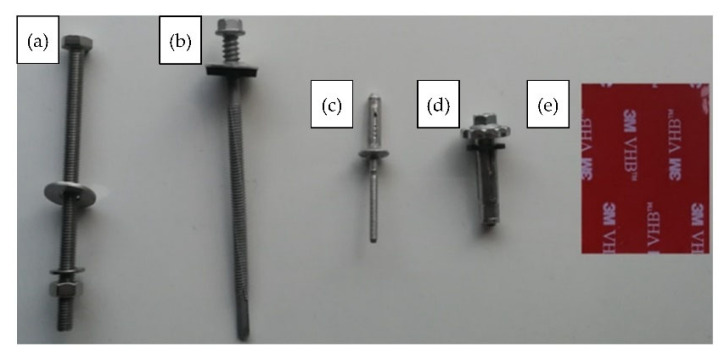
Connectors used to join the thin-walled beam and the sandwich panel (description in the text): (**a**) bolt (B), (**b**) self-drilling fastener dedicated for sandwich panels (F), (**c**) pulled blind rivet (BT), (**d**) tightened blind rivet (FB), (**e**) acrylic tape (TL, TP).

The thin-walled steel beam with a Z-section was fastened to the sandwich panel at an axial distance of 150 mm from its free edge. The dimensions of the thin-walled beam cross section were equal to 18 × 39 × 100 × 45 × 18 mm with constant walls thicknesses of 1.5 mm. The properties values of the thin-walled beam material were determined on the basis of laboratory tests [18,28]. Young’s modulus was equal to 198 MPa, the value of the upper yield strength was equal to 348.87 MPa, and the value of the ultimate tensile strength was equal to 402.72 MPa. The following five types of connectors to the authors’ knowledge can be used to connect the thin-walled section to the sandwich panel:

- Bolts (B) fully threaded with a diameter equal to 6 mm and length equal to 100 mm; see Figure 2a;
- Self-drilling fasteners for fastening sandwich panels to steel construction (F) with a diameter equal to 6.3 mm and length equal to 110 mm; see Figure 2b;
- Pulled blind rivets (BT) (threefold aluminium blind rivets of a diameter equal to 4.75 mm and clamping arm’s length measured after pulling equal to 6 mm); see Figure 2c;
- Tightened blind rivets (FB) (fourfold steel/aluminium blind rivets of a diameter equal to 7.80 mm and clamping arm’s length measured after pulling equal to 10 mm); see Figure 2d;
- Double-sided acrylic foam tape (TL—applied continuously, TP—applied pointwise) with thickness equal to 1.5 mm and width equal to 38 mm; see Figure 2e.

Sandwich panels used in structural engineering applications are usually attached to the supporting structure by three fasteners per width. Therefore, in the experiment, three mechanical connectors per width (B, F, BT, and FB) were used, that is, in the middle of the beam and 500 mm on the left and on the right from the middle point. In the case of acrylic tape, two methods of joining were used. In the first method of joining (TP), three short sections of tape measuring 38 × 50 mm were used. In the second method (TL), the tape was glued linearly along the entire length of the thin-walled beam. Double-sided acrylic foam tape, used in the experiments, may be used to bond a variety of materials, such as aluminium, steel, glass, plastics, painted, or powder-coated surfaces.

**Figure 3:**
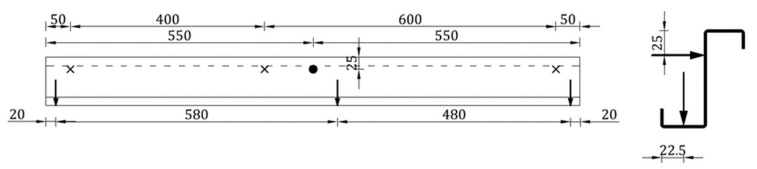
Position of the force and inductive displacement transducers along the thin-walled beam (description in the text).

In the experiment, the Z beam-free flange was subjected to a horizontal load perpendicular to the central beam axis (to the beam web). The load was applied by a loading cell with a speed of 5 mm/min. The exact position of the loading cell is described in Figure 3 through a bold dot. Force was measured using a force transducer (U93) of 10 kN capacity of 0.5 class (it gives the accuracy of force measurement equal to 0.05 kN). During the test, vertical and horizontal beam displacements were measured at three points: in the middle of the beam span and at the outer edges of the beam. The precise position of the inductive displacement transducer with nominal displacements of 50 mm and 100 mm (WA 50 and WA 100) is depicted in Figure 3.

**Figure 4:**
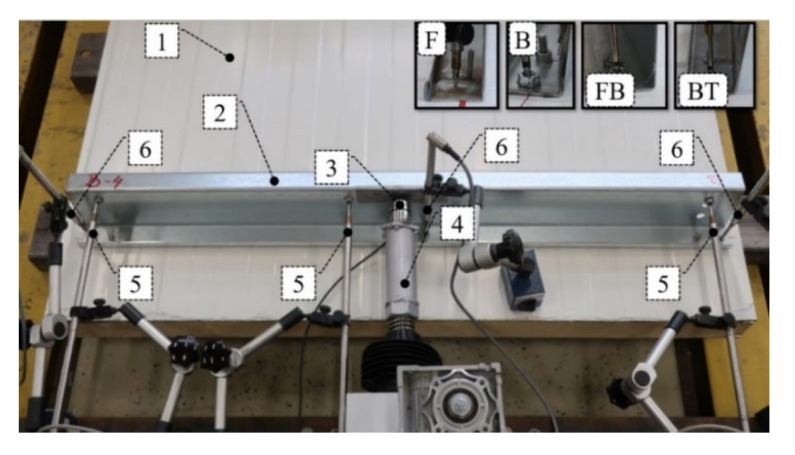
View of a test bed (description in text).

In Figure 3, ‘×’ represents the position of horizontal displacement transducers, vertical arrows represent the position of vertical displacement transducers, and ‘bold dot’ represents the position of the horizontal force transducer. Figure 4 presents the view of the test bed where **1** represents a sandwich panel with polyisocyanurate foam core and steel lightly profiled facings, **2** represents a thin-walled Z beam, **3** represents force transducer U93, **4** represents the electromechanical actuator (assembled by Archimedes Ltd., Toruń, Poland), **5** represents the inductive displacement transducers WA 100 (range 100 mm), **6** represents the inductive displacement transducers WA 50 (range 50 mm), **F** represents the typical self-drilling fastener, **B** represents the bolt, **FB** represents the tightened rivet, and **BT** represents the pulled blind rivet. It is worth noting that the innovative use of pulled blind rivets was presented in References [29,30] where laboratory and numerical analyses of the beam-to-column connection in cold formed steel frames were carried out.

### 2.2. Experimental Investigation

**Figure 5:**
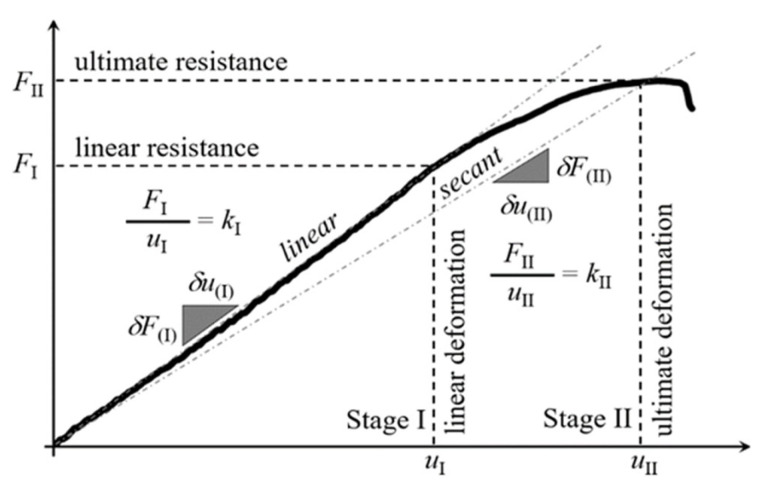
Connections’ parameters.

The connection methods considered in the paper can be divided into three groups. The first group is represented by mechanical connectors that penetrate all layers of the sandwich panel: self-drilling fasteners (F) and bolts (B). The second group is represented by mechanical connectors: tightened blind rivets (FB) and pulled blind rivets (BT) that are attached only to one sandwich panel facing. Finally, the third group represents the non-penetrating connection method using continuous (TL) and pointwise (TP) tape arrangements. Each of the connection methods was investigated experimentally. The testbed setup was described in Section 2 and is depicted in Figure 4. The equilibrium load-displacement paths, obtained for each type of connection, basically represent the two-stage response: linear and nonlinear. The linear response was determined using the iterative linear regression formula. The ultimate point is defined as a point in which the derivative of the load-displacement curve is less than zero. The equilibrium load-displacement paths obtained provide information on the linear and ultimate resistance, the linear and ultimate deformation capacity, and the linear and secant stiffness, which are graphically depicted in Figure 5.

## 3. Results

**Figure 6:**
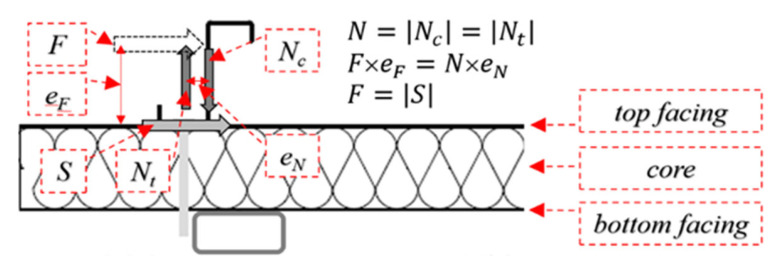
General scheme of the forces in the investigated connections.

Please note that the mechanical response of penetrating connectors also consists of the initial response, which refers to the initial loading phase (up to 0.1 kN). It arises from the initial stiffness of the section when the connection is tightly connected with the sandwich panel from the beginning of the loading process (F, B). In all figures that present the equilibrium load-displacement curves from the experiments, the vertical line represents the boundary between the linear (I) and non-linear (II) stages. Additionally, in the figures mentioned, the thick black continuous lines represent the mean value of the individual results of the connector type considered. The thin grey lines represent the load-displacement paths recorded during laboratory experiments. Note that in the experiment, the horizontal force (*F*) applied in the middle length of the element (Figure 3 and Figure 4) is transferred by the support conditions of the thin-walled element as a pair of normal forces (*N*) to the surface of the sandwich panel and horizontal forces (*S*). The shear resistance of the connectors transfers the top facing of the horizontal force to the sandwich panel. The normal force, located along the rotation line, is directed into (*N_c_*) the sandwich panel facing while the other one, localized along with the connectors, is directed outward (*N_t_*); see Figure 6. As presented in Figure 6, the force distribution is general for all investigated connection types.

### 3.1. Failure Scenarios and Equilibrium Load Displacement Paths

#### 3.1.1. Connectors Penetrating Whole Sandwich Panel Depth

**Figure 7:**
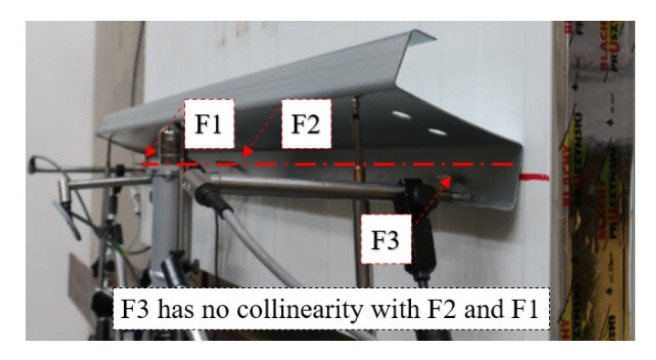
Lack of the collinearity of the self-drilling fasteners (F).

**Self-drilling (F) fasteners** are commonly used in civil engineering applications to connect thin-walled elements to sandwich panels. The use of self-drilling fasteners was also recently investigated in [26] where the test bed was used for the double lap shear test. This type of connection does not require predrilling of the hole and therefore is easy and fast to install. However, during installation, the substructure is below the sandwich panel and thus is not visible to the cladding fitter. This leads to the problem of achieving collinearity of the fasteners during sandwich panel installation; see Figure 7.

**Figure 8:**
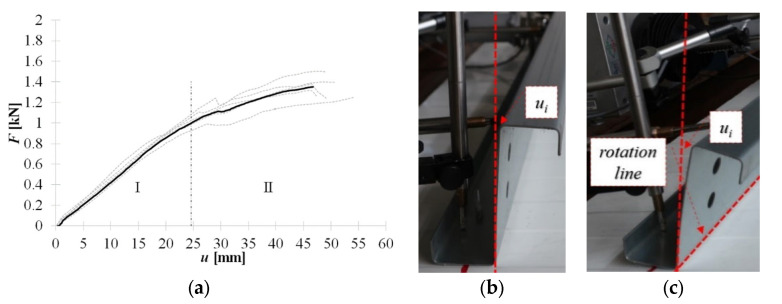
Self-drilling fasteners (F): (**a**) equilibrium load- displacement paths, (**b**) initial (up to 0.05 kN) response, (**c**) linear response (stage I).

In Figure 8a, the equilibrium load-displacement paths of self-drilling fasteners (F) are presented. It was observed during the experiments, that up to *F* ≈ 0.05 kN the load is compensated by the stiffness of the cross-section. It is manifested as an elastic bow deformation of the web along with its depth without cross-section rotation; see Figure 8b. With increasing load, the section starts to rotate. The rotation line is located on the fold between the web and the bottom flange, see Figure 8c. The observed web bow of the cantilever-buckling shape was kept to the end of the linear part of the equilibrium path (stage I). Thus, the initial angle between the bottom flange and the web remains constant during the linear stage (I).

**Figure 9:**
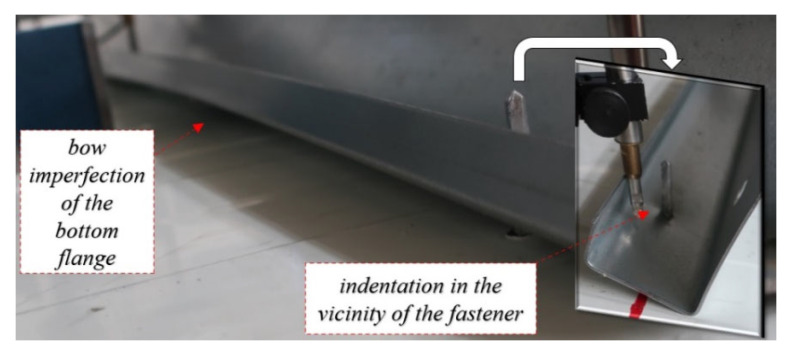
Failures in the nonlinear stage of the kinematical response of the connection (stage II) using self-drilling fasteners (F).

An additional increase in the load (stage II—non-linear behaviour) increases the deformation of the web bow and the section rotation. Rotation of the cross section results in an indentation of the bottom flange in the vicinity of the fasteners (see Figure 9) and its slip over the fastener thread. Additionally, the cross-section rotation is compensated by the bottom flange bow deformation depicted in Figure 9. It should be noted that the position of the connectors with respect to the cross-sectional web is important in preventing rotation. The closer the fasteners are to the web, the higher the cross-sectional rotation is observed.

**Figure 10:**
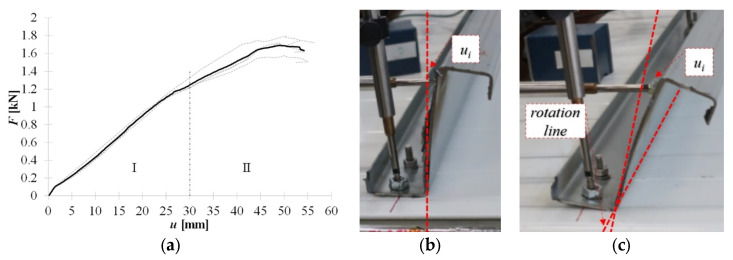
Self-drilling fasteners (F): (**a**) equilibrium load-displacement paths, (**b**) initial (up to 0.05 kN) response, (**c**) linear response (stage I).

Figure 10a shows the equilibrium load-displacement paths of the connection between the thin-walled beam and sandwich panel made with the **bolts** with a nut and two steel washers. This connection requires pre-drilling of all layers of the sandwich panel (both facings and core) and the bottom flange of the thin-walled section. Thus, the potential problem of collinearity is easier to eliminate. It is worth noticing that this connection is characterized by a similar kinematical response to the self-drilling fasteners, i.e., the same stages can be distinguished. That is, it was observed that initially (up to *F* ≈ 0.1 kN) the section compensates the load by an elastic bow deformation of the web along with its depth without cross-section rotation; see Figure 10b. 

**Figure 11:**
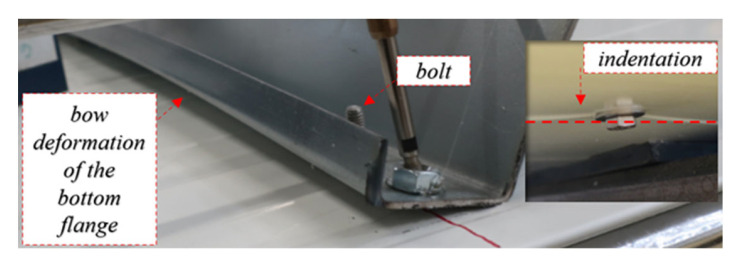
Failure mechanisms in stage II in connection using bolts: indentation at the bottom facing and bow deformation of the bottom flange.

An increase in the load (above stage I) leads to an increase in the rotation of the cross section. The use of bolts with nuts prevents the bottom flange of the beam from being separated from the top facing of the sandwich panel. However, at stage II, the bow deformation of the bottom flange is also observed; see Figure 11. Additionally, the washer between the nut and the bottom flange prevents local indentation in the bottom cross section flange, which was observed in the case of self-drilling fasteners. However, the uplift force *N_t_* (see Figure 6) causes the indentation of the bottom facing in the vicinity of the head and the unscrewing of the nut, see Figure 11. 

#### 3.1.2. Connectors Attached to One Sandwich Panel Facing—Blind Rivets

**Figure 12:**
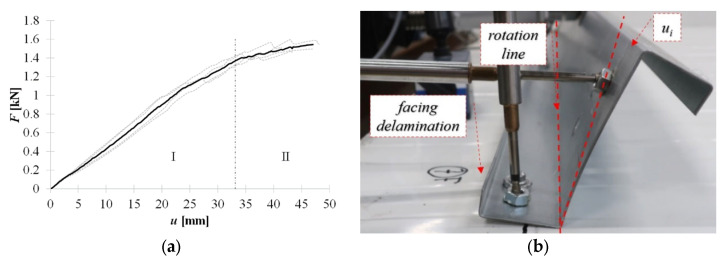
Tightened blind rivet: (**a**) equilibrium load-displacement paths, (**b**) connection behaviour in stage I.

The installation of both types of blind rivets considered requires predrilling of the holes. Thus, similarly to the bolts, the problem with their collinearity is easier to eliminate. After predrilling, the rivets are inserted into the holes, and the bottom flange of the cross section and one sandwich panel facing are tied. Note that in this case, the core layer is not penetrated; thus, no point thermal bridges are created. The diameter of the tightened and pulled blind rivets, as well as their length, varies, and thus, their results are presented separately. However, general conclusions can be drawn, namely, that the blind rivet clamping arm significantly influences connection strength, deformation capacity, and failure mechanisms. Figure 12a shows the equilibrium load-displacement paths of the connection realized by the **tightened blind rivets**. The initial stiffness of the cross section, which occurred with fasteners and bolts, was not observed in this connection. It means that from the beginning, the rotation of the cross section and the web bow deformation occurred; see Figure 12b.

**Figure 13:**
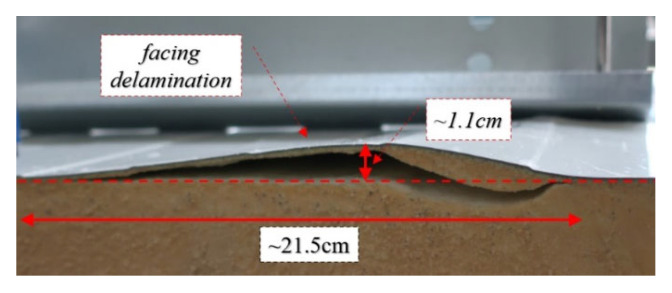
Tightened blind rivet–facing delamination in stage II.

Furthermore, facing delamination was observed on the extension of the blind rivet line (perpendicular to the length of the thin-walled beam). The further load increase increases cross-section rotation and facing delamination that reaches the edge of the sandwich panel, see Figure 13. 

**Figure 14:**
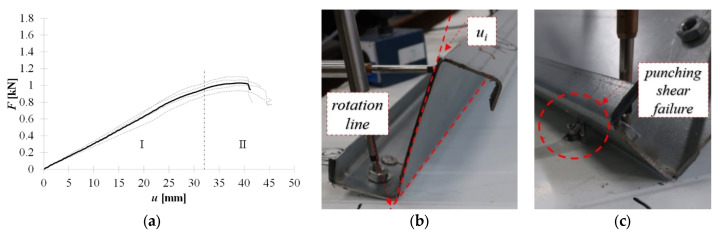
Pulled blind rivet: (**a**) equilibrium load-displacement paths, (**b**) cross-section rotation at stage I, (**c**) punching shear failure at stage II.

Figure 14a shows the equilibrium load-displacement paths of the connection realized by **pulled blind rivets**. In the case of these connectors, it was observed that the bow deformation of the cross-sectional web was negligible, see Figure 14b. The deformation of the connection is manifested by the cross-sectional rotation which, in stage II, leads to the punching shear failure. The punching shear failure mechanism is connected to the plastic deformation of the clamping arms of the blind rivet.

#### 3.1.3. Non-Penetrating Connectors

**Figure 15:**
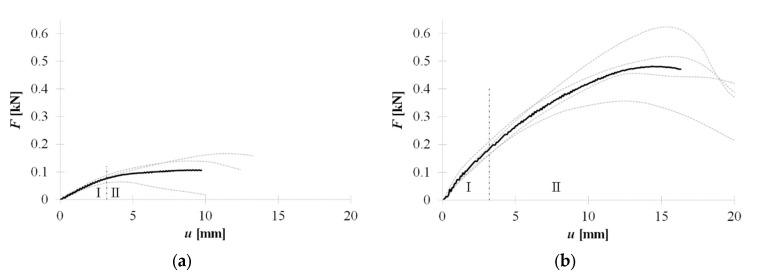
Equilibrium load-displacement paths of non-penetrating connectors: (**a**) pointwise tape (TP), (**b**) continuous tape (TL).

The last method of connection is performed with double-sided acrylic tape. In Figure 15a,b the equilibrium load-displacement paths of the pointwise (TP) and continuous tape (TL) arrangement, respectively. Please note that the ultimate strength, stiffness, and deformation capacity of the connection achieved with the use of tape applied pointwise is significantly lower than with the use of tape applied tape.

**Figure 16:**
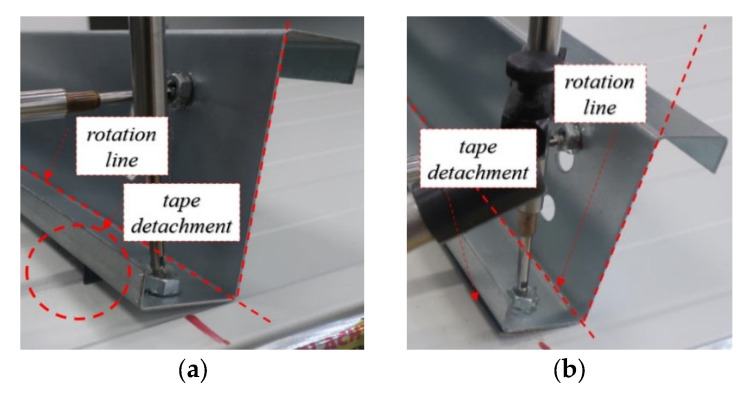
Cross-section rotation without bow web deformation: (**a**) pointwise tape (TP), (**b**) continuous tape (TL).

In the case of pointwise tape and continuous tape, the cross section remains straight during stage I and stage II, that is, no bow deformation of the cross section web was observed; see Figure 16a,b, respectively. It was due to the very small capacity of the connection. In this case, only the tape detachment governed the load-displacement behaviour of the connection.

## 4. Discussion

In Table 1, data collected from all tests are presented. These data represent the load-horizontal displacement relation of the middle point located in the beam web. The parameters are illustrated graphically in Figure 5. Nevertheless, for the record in Table 1, the following parameters are listed:

- *F*_I_ and *F*_II_ represent linear and ultimate resistance, respectively;
- *u*_I_ and *u*_II_ represent linear and ultimate deformation capacities, respectively;
- *k*_I_ and *k*_II_ represent linear and secant stiffness, respectively;
- *A*_I_ and *A*_II_ represent the area below the curve for linear and nonlinear part, respectively.

Resistances and horizontal deformations represent the mean values for each group of connectors (*n* represents the statistical sample size). Please note that, in the case of nonmechanical connectors (tapes), the scatter of the results is significant; therefore, they should not be considered in this type of usage.

**Figure 17:**
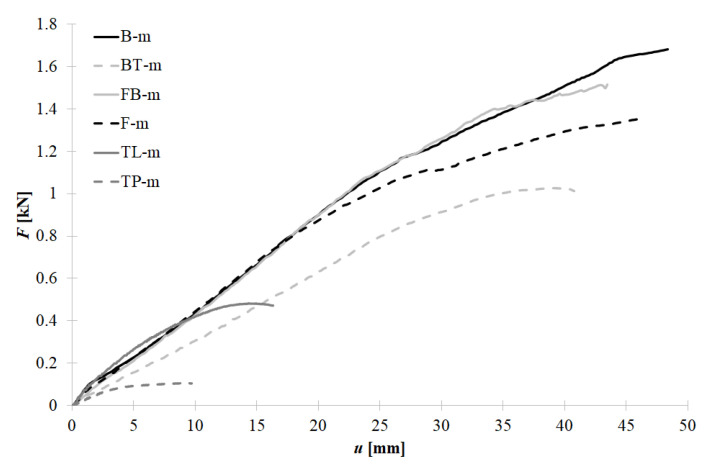
Mean equilibrium load-displacement paths of the various method of connection between thin-walled beam and sandwich panel.

In Table 1, AI and AII represent the area below the linear and nonlinear parts of the load-displacement curves. These areas can be interpreted as the strain energy capacity of the connection for its linear and nonlinear mechanical response. In Figure 17, the mean representations of each connection type are plotted. One can observe that the bolt connection (B) and tightened blind rivets connection (FB) are characterized by exact behaviour in stage one (linear response) and comparable in stage 2 (nonlinear response). The typically used self-drilling fasteners (F) are characterized by the same linear stiffness as bolts (B) and tightened blind rivets (FB) but are also characterized by 20% lower ultimate resistance and secant stiffness. Nevertheless, the deformation capacity of bolts (B), tightened blind rivets (FB), and self-drilling fasteners (F) is comparable. The mechanical response of the pulled blind rivet (BT) connection is significantly smaller than the response of other mechanical connectors. This is because its clamping arm’s length and diameter are smaller than in the case of the tightened blind rivet. This leads to a failure by pulling the rivets from the facing. Non-mechanical connectors—tapes—are characterized by their lowest resistance and deformation capacity.

**Figure 18:**
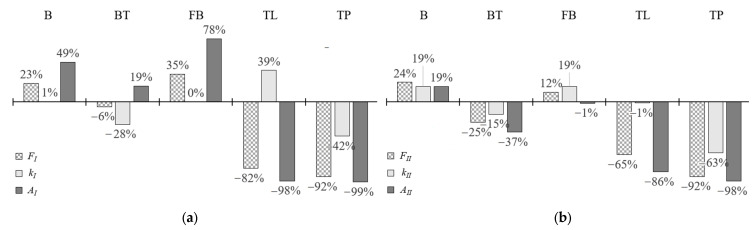
Change of the resistance (*F_i_*), stiffness (*k_i_*), and strain energy capacity (*A_i_*) concerning the self-drilling fasteners (F): (**a**) linear stage, (**b**) non-linear stage.

Due to the fact that self-drilling fasteners are used in the case of civil engineering applications, the results for this connector were considered as reference results for the other methods of connecting sandwich panels with thin-walled beams. In Figure 18a,b, the changes of three selected parameters for the linear and non-linear stages are depicted, respectively. This comparison sustains the conclusions defined above, i.e., that the bolts (B) and tightened blind rivets (FB) are characterized by higher resistance and deformation capacity than self-drilling fasteners.

## 5. Conclusions

The laboratory results presented in the paper, give quantitative information on the possible usage of the different connectors to connect the thin-walled members to sandwich panels. These results can be considered as a starting point when full-scale laboratory tests are being prepared in the area of stabilization of the thin-walled members by sandwich panels. The research was limited to a core layer material, i.e., polyisocyanurate foam core (PIR foam). However, in the case of the through-drilling connectors (fasteners, bolts) and the tapes, the failure mechanisms were not connected with the mechanical properties of the core layer, but rather with its thickness. Therefore, the results of these types of connectors could be extended to other core layer materials, for example, styrofoam or mineral wool. It cannot be assumed only for blind rivets because the observed failure mechanism was related to the core layer delamination. In that case, further considerations could be directed to the use of analytical solutions described in [31,32].

## Figures and Tables

**Table 1:** Data from laboratory test.

Parameter	Unit	F	B	BT	FB	TL	TP
*F*_I_	[kN]	1.02 ± 0.06	1.25 ± 0.06	0.95 ± 0.07	1.37 ± 0.05	0.18 ± 0.02	0.08 ± 0.01
*u*_I_	[mm]	24.63 ± 3.73	30.03 ± 1.44	32.08 ± 2.20	33.13 ± 1.83	3.20 ± 0.17	3.20 ± 0.07
*k*_I_	[kN/m]	41.3	41.6	29.9	41.3	57.5	23.9
*A*_I_	[kNm]	0.0135	0.0201	0.0161	0.0240	0.0003	0.0001
*F*_II_	[kN]	1.35 ± 0.09	1.68 ± 0.09	1.01 ± 0.07	1.52 ± 0.05	0.47 ± 0.12	0.11 ± 0.05
*u*_II_	[mm]	46.18 ± 3.64	48.16 ± 1.86	40.75 ± 0.80	43.47 ± 2.37	16.32 ± 2.00	9.72 ± 4.11
*k*_II_	[kN/m]	29.3	34.9	24.9	34.9	28.9	10.9
*A*_II_	[kNm]	0.0396	0.0471	0.0248	0.0390	0.0054	0.0008
*n*	[–]	5	4	4	4	4	3

## Data Availability

Not applicable.
